# The Impact of Surgery on Quality of Life in Hidradenitis Suppurativa: Results from a Prospective Single-Center Study

**DOI:** 10.3390/life15050769

**Published:** 2025-05-12

**Authors:** Lennart Ocker, Nessr Abu Rached, Anna Koller, Carolin Frost, Riina Käpynen, Falk G. Bechara

**Affiliations:** International Centre for Hidradenitis Suppurativa/Acne Inversa (ICH), Department of Dermatology, Venereology and Allergology, Ruhr-University Bochum, 44791 Bochum, Germanyfalk.bechara@klinikum-bochum.de (F.G.B.)

**Keywords:** hidradenitis suppurativa, dermatologic surgery, quality of life, inflammation

## Abstract

Hidradenitis suppurativa (HS) is a chronic inflammatory skin disease that severely impairs quality of life. Treatment typically involves a patient-oriented combination of medical therapies, surgery, and lifestyle modifications. However, data on the impact of surgical treatments on quality of life remain limited. This prospective monocentric study aimed to evaluate the effect of wide surgical excision in patients with moderate to severe HS (Hurley stage II/III) who were naïve to systemic biologic treatments. Between March 2017 and November 2022, 82 patients (51% female; 80% Hurley II, 20% Hurley III) underwent major surgical excision. Assessments were performed before surgery and at three and six months postoperatively. The primary endpoint was the change in Dermatologic Life Quality Index (DLQI); secondary endpoints included changes in pain (NRS-11) and disease severity scores. DLQI improved from 11.7 at baseline to 8.3 at three months and 4.7 at six months (*p* < 0.001). Pain scores and the modified Hidradenitis Suppurativa Score (mHSS) also significantly decreased (*p* < 0.001). In conclusion, major surgery significantly improved quality of life and pain in HS patients, confirming its essential role in a multimodal treatment approach. Patient-reported outcome measures are crucial for assessing treatment efficacy in HS.

## 1. Introduction

Hidradenitis suppurativa (HS) is a chronic inflammatory skin disease resulting in recurrent inflammatory lesions and subsequent fibrosis and tunnel formation, predominantly in intertriginous body areas [1]. Incidence rates vary between 0.1 and 1.8% and individual treatment depends on multiple factors, such as the extent of the disease, skin lesions, inflammatory load, and patient preference [2,3].

HS is associated with a high burden of disease and compromises the social and professional life of affected patients [4]. Patients experience chronic pain and fear of stigmatization, leading to social isolation [5,6]. Through its predominant manifestation in intertriginous areas, the disease has also an impact on the sexual life of patients [7].

The treatment of HS patients is dependent on multiple factors, such as the types of lesions, inflammatory activity, patient preference, and comorbidities, and it should be managed in an individualized patient-oriented approach [8]. Often, a combined treatment approach using medical and surgical treatment modalities is needed [8].

In recent years, intensified research activity has led to the identification of new promising molecular targets for medical therapy. Currently, with bimekizumab, secukinumab and adalimumab, there are three biologic monoclonal antibodies available for HS treatment [9,10,11]. These targeted treatments have been shown to reduce inflammatory activity in affected patients. However, there are only limited data about their effects on patient-reported outcome measures (PROMs) [11,12,13].

Surgery marks another important pillar in the treatment of HS and is mainly indicated in advanced cases with irreversible tissue damage, such as sinus tracts, scars, and contractions [8,14]. There are contradicting data on the impact of surgical interventions on the quality of life of HS patients. Therefore, we sought to investigate the impact of wide surgical excision on the quality of life of HS patients in a prospective study.

## 2. Materials and Methods

In this single-center prospective study, patients with moderate to severe HS, naïve to systemic biological treatment, undergoing wide surgical excision between March 2017 and November 2022 at the International Center for Hidradenitis Suppurativa/Acne inversa, University Hospital Bochum, were included. Diagnosis of HS and indication for surgery were determined by two experienced dermatologists following the Dessau criteria [15]. Only patients with complete follow-up data were included in this study.

Prior to surgery, all patients underwent a baseline examination (V0), in which HS severity (Hurley stages and modified Sartorius score) and patient-reported outcome parameters (pain score via the Numerical Rating Scale (NRS-11) and Dermatologic Life Quality Index (DLQI)) were recorded. The DLQI is a validated, dermatology-specific quality of life instrument consisting of 10 questions covering symptoms, feelings, daily activities, and social interactions [16]. Surgery was performed under general anesthesia followed by either secondary intention healing or split-thickness skin grafting. Following surgery, regular follow-up visits were scheduled after three (V3) and six months (V6), respectively.

The primary endpoint was the change in life quality, measured with DLQI, after surgical intervention [16]. Secondary endpoints were the change in disease severity scores (Hurley staging system and Hidradenitis Suppurativa Score (HSS)) and reduction in pain (NRS-11) [17,18,19].

Statistical analysis was performed with IBM SPSS Statistics version 30.0.0 (IBM corporation, Armonk, NY, USA). Parametric repeated-measure ANOVA with Huynh–Feldt correction was conducted for statistical analysis of DLQI changes. Postoperative changes in HSS and NRS-11 were calculated with non-parametric Friedman rank tests. Statistical differences between individual measuring points were calculated with Bonferroni–Holm-adjusted post hoc analysis. Subgroup analyses were performed with covariate analysis (ANCOVA). Statistical significance was considered as *p* < 0.05. No formal sample size calculation was performed prior to study initiation, as the primary aim was to prospectively collect real-world data on all eligible patients undergoing wide surgical excision for moderate to severe hidradenitis suppurativa at our center within a defined period (March 2017 to November 2022). The final sample of 82 patients reflects the complete cohort meeting the inclusion criteria and completing follow-up visits.

The study was conducted in accordance with the Declaration of Helsinki and approved by the ethics committee of the Ruhr-University Bochum (protocol code 5076–14 and date of approval 30 June 2014).

## 3. Results

Between March 2017 and November 2022, 82 patients were included in this study (51.2% female, mean age at surgery: 37.5 (SD ± 12.7) years). Overall, 60.1% of the patients (*n* = 50) were active smokers and 32.9% (*n* = 27) reported a positive family history for HS. The mean BMI was 30.21 (SD ± 6.7) kg/m^2^. Wound healing by secondary intention was pursued in 78.0% of patients (*n* = 64), while 22.0% of patients (*n* = 18) received split-thickness skin grafting following radical excision. Detailed information regarding patient characteristics is shown in Table 1.

Prior to surgery, 80.5% of patients (*n* = 66) were categorized as Hurley stage II and 19.5% (*n* = 16) as Hurley stage III. Six months after surgery, 24.4% (*n* = 20) of patients had no HS lesions and were therefore considered as Hurley stage 0, while 54.9% (*n* = 45) were categorized as Hurley stage I, 18.3% (*n* = 15) were categorized as Hurley stage II, and 2.4% (*n* = 2) were categorized as Hurley stage III, respectively.

### 3.1. Impact of Surgery on Quality of Life

**Figure 1:**
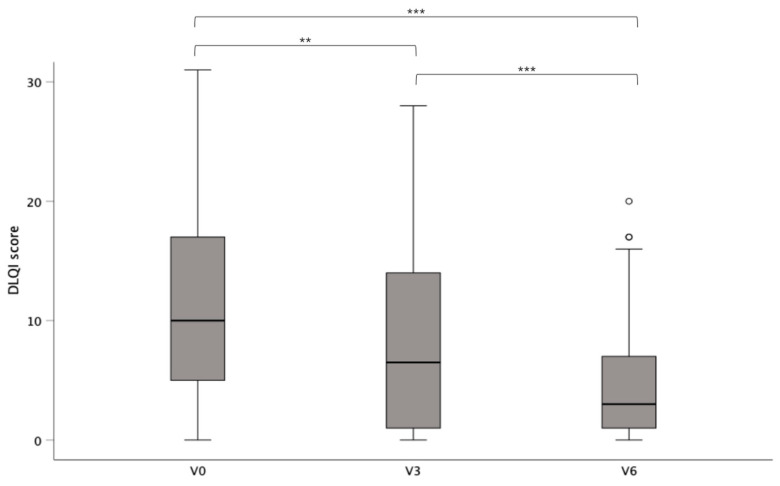
Mean DLQI scores (±SD) of patients before (V0) and 3 and 6 months after surgery (V3; V6).; *p* < 0.01 (**); *p* < 0.001 (***).

Seventy-three of the patients in our study had a complete record of DLQI scores, and the mean DLQI score was 11.66 (SD ± 8.28) prior to surgery. Following surgery, DLQI scores were reduced to 8.26 (SD ± 7.37) and 4.65 (SD ± 4.87) after three and six months, respectively. Forty-one patients (56.2%) reported DLQI improvements of ≥5 points from baseline after six months. Repeated-measure ANOVA with Huynh–Feldt correction revealed statistically significant differences between the measuring points (F(1.84;134.64) = 32.24; *p* < 0.001, partial η^2^ = 0.3). A Bonferroni-adjusted post hoc analysis revealed significant differences between DLQI scores at the different measuring points (Figure 1).

### 3.2. Impact of Surgery on Disease Severity

**Figure 2:**
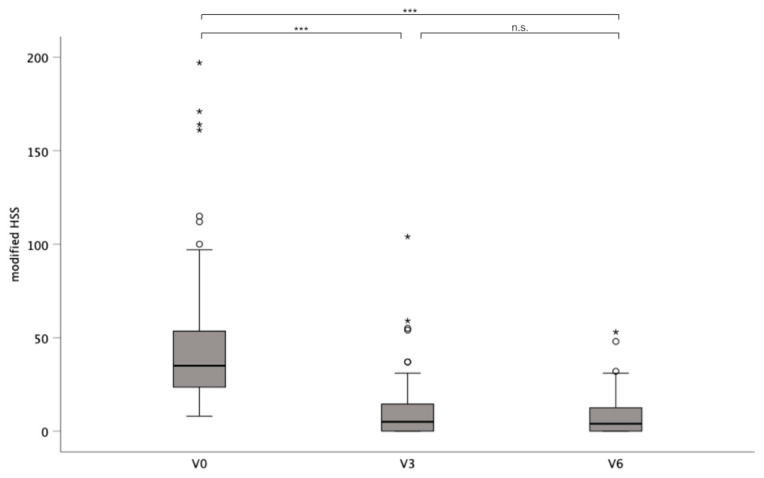
Mean modified Hidradenitis Suppurativa Score (mHSS) (±SD) of patients before (V0) and 3 and 6 months after surgery (V3; V6). *p* < 0.05 (*); *p* < 0.001 (***).

The mean HSS was 50.3 (SD ± 49.3) at baseline and 10.6 (SD ± 16.8) and 8.61 (SD ± 10.8) after 3 and 6 months, respectively (*p* < 0.001). A post hoc analysis with Bonferroni correction revealed significant differences between baseline and after 3 and 6 months, respectively (*p* < 0.001). No significant changes were found between HSSs at 3 and 6 months (*p* = 0.75) (Figure 2).

### 3.3. Impact of Surgery on Pain

**Figure 3:**
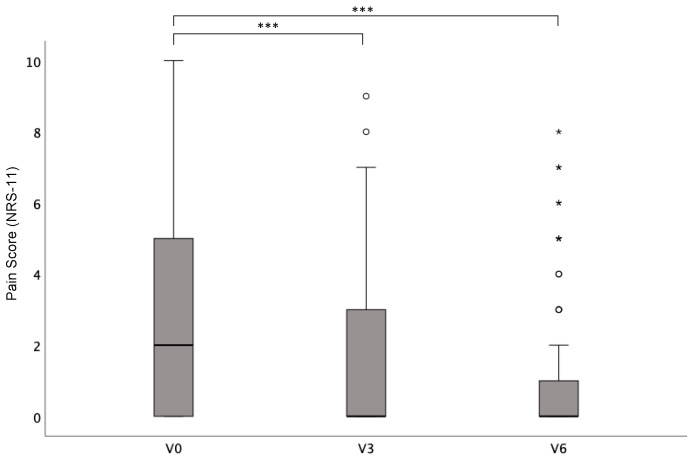
Mean patient-reported pain (measured with NRS-11) (±SD) before (V0) and 3 and 6 months after surgery (V3; V6). *p* < 0.05 (*); *p* < 0.001 (***).

Pain was evaluated with the numerical rating scale (NRS-11) and mean NRS scores were 3.01 (SD ± 3.04) at baseline compared to 1.46 (SD ± 2.42) at 3 months after surgery and 1.07 (SD ± 2.02) at 6 months after surgery. Non-parametric Friedman rank testing was significant (*p* < 0.001) and a post hoc analysis with Bonferroni correction revealed significant differences between the baseline NRS score and 3 and 6 months postoperatively (*p* < 0.001). No significant differences were found between pain scores at 3 and 6 months after surgery (*p* = 0.45) (Figure 3).

### 3.4. Secondary Intention Healing vs. Skin Grafting

The effects of split-thickness skin grafting on patient-reported outcome parameters were investigated in a subgroup analysis. Of a total of 82 patients in our study collective, 18 patients (22%) received STSG in the postoperative course, while secondary intention wound healing was established in 64 patients (78%).

The effect on quality of life was investigated using a linear univariate covariate analysis, comparing DLQI scores at baseline and after 6 months in both groups. Mean DLQI scores in the STSG group were 8.14 (SD ± 5.25) and 4.93 (SD ± 4.75) after 6 months, compared to 12.48 (SD ± 8.67) and 1.03 (SD ± 1.97) in the secondary wound healing group. Univariate analysis (ANCOVA) revealed a significant difference between both treatment groups (F = 25.48; *p* < 0.001).

NRS-11 scores were evaluated after three and six months postoperatively. The mean pain scores were 3.82 (SD ± 3.40), 2.06 (SD ± 2.99), and 0.94 (SD ± 2.22) in the STSG group compared to 2.80 (SD ± 2.92), 1.30 (SD ± 2.25), and 1.11 (SD ± 1.99) in the secondary wound healing group. Univariate testing revealed no significant differences between treatment groups after three months (F = 0.778; *p* = 0.380) and six months (F = 0.225; *p* = 0.637).

## 4. Discussion

Hidradenitis suppurativa is associated with a pronounced impairment of the quality of life of affected patients. Several factors, such as comorbidities, unemployment, and disease severity, can further amplify the disease’s impact on quality of life and complicate the treatment of these patients [20]. Besides objective treatment outcome parameters such as disease severity scores, quality of life has emerged as a significant patient-reported outcome parameter in clinical trials, and medical treatments have also been evaluated based on their impact on quality of life [21]. Different measurement tools have been described to assess quality of life in patients with HS [20]. The dermatologic life quality index is a widely used self-reported screening tool for dermatologic patients, consisting of ten questions. Through its simplicity and its fast applicability, DLQI is the most commonly used screening tool for quality of life; however, it is not specific for HS [16,22]. Several HS-specific health-related quality of life instruments like the Hidradenitis Suppurativa-Quality of Life (HS-QoL) and the Hidradenitis Suppurativa Burden of Disease (HS-BOD) have been developed in recent years; however, their clinical application is limited [23,24,25,26].

In the PIONEER I and II studies evaluating adalimumab in patients with moderate to severe HS, only slight improvements of quality of life were observed within the 12 week treatment period [9,12]. In the off-label extension phase of these studies, significant improvements in DLQI and pain scores were reported [27]. The interleukin-17 antibody secukinumab was evaluated in two recent phase III clinical trials. Pooled data from the SUNSHINE and SUNRISE trials showed significant DLQI improvements, defined as DLQI reduction of at least five points from baseline, in 42.5% and 47.1% of patients receiving secukinumab every 2 and every 4 weeks, compared to 30.7% in the placebo group [10]. Bimekizumab, a humanized monoclonal anti-IL17A/F antibody has recently been investigated in a phase III study in HS patients and showed rapid responses to therapy as well as significant improvements in patient-reported outcomes such as quality of life [11]. In our study, a DLQI reduction of at least five points was reached in 56.2% of patients after six months. However, it has to be noted that only patients naïve to biologic treatments were included in this study, and even higher responses could be anticipated by a combination of adequate surgery and biologic treatments.

According to current guidelines, surgical treatment of HS is generally indicated in advanced disease with irreversible tissue destruction, fibrosis, and tunnel formation [28,29,30]. The selection of surgical treatment depends on various factors, such as the chronicity and extent of the disease, the affected site, the presence of long-standing lesions, and inflammatory activity [28]. While radical surgery may lead to long-term remission in selected patients, hidradenitis suppurativa is generally considered a chronic relapsing disease [8]. The physician’s experience and attitude toward HS surgery also play a key role in treatment decisions [31]. Surgical treatment in HS ranges from procedural treatments (e.g., laser) and minor surgery (e.g., incision and drainage and deroofing) to major surgery (e.g., wide local excision) [8]. Surgical outcomes can vary depending on disease extent, patient factors, and surgical expertise. Despite surgical treatment options having been described extensively in HS patients, there are only a small number of studies on their effectiveness, demonstrating contradicting data on their impact on the quality of life of affected patients. This may be partly explained by the lack of standardization of surgical interventions in HS [1].

In a cross-sectional study with 500 HS patients, 40% reported a very large impairment in their quality of life [32]. However, no significant association with surgical or medical treatment was found in this study [32]. Grimstad et al. compared the effects of medical and surgical treatments in 255 patients from the Scandinavian HS Registry. Overall, 55% of patients reached a significant improvement in the DLQI score and 49.7% reported a reduction in pain after surgery, compared with 28% and 31% after medical treatment [33].

In a retrospective questionnaire-based patient survey, most patients were satisfied with their surgical results and 82.6% would recommend surgery to other patients. The retrospective quality of life score assessed with a 0–10 scale improved from 5 points prior to surgery to 8.4 points after surgery; however, DLQI was not evaluated [34]. In another retrospective questionnaire-based study, 255 patients with moderate to severe HS were interviewed after surgical intervention. Overall, 97% of patients reported restrictions in their everyday life and around 60% of patients reported restrictions in their professional life [35]. Quality of life after surgery was not evaluated; however, a majority of patients rated their postoperative outcomes as satisfying and recommended surgery as a treatment option [35].

A prospective single-center study evaluated different patient-reported outcome measures in 40 patients undergoing surgical treatment and found no significant differences in DLQI within the 6-month follow-up period. These findings were explained by prolonged wound healing [36].

Posch et al. reported a significant reduction in the DLQI after wide local excision in 74 Hurley stage II patients, and 70% were satisfied with their postoperative outcome. However, the retrospective design of the study was a major limitation [37]. In a single-center prospective study with 55 HS patients, a significant reduction in DLQI and pain score was achieved through wide local excision followed by secondary intention healing [38].

In the last decade, the treatment of HS has evolved into a multimodal patient-oriented approach comprising targeted systemic therapies for active inflammation and surgical treatment for irreversible tissue damage. However, in a clinical real-world setting, there are distinct situations where systemic anti-inflammatory therapies are not indicated or are eventually declined by the patient. In these cases, adequate surgical therapy can provide stabilization and prevent disease progression. To our knowledge, this is currently the largest prospective study focusing on the impact of surgical treatment on the quality of life in biologic-naïve HS patients. While our findings highlight the role of surgery as a cornerstone in the treatment of moderate to severe HS, the integration of surgical and medical treatments—including biologic agents—is essential for achieving disease control in many patients [29,39,40]. Particularly in cases of persistent inflammation or extensive disease, a multimodal treatment strategy may offer synergistic benefits [41,42]. Further research should define how best to combine biologics and surgery, including optimal sequencing and patient selection.

In this study, only patients with advanced HS that underwent wide excision of affected areas were included. A significant improvement in the DLQI score was observed within the first three months after surgical treatment and further reductions were achieved in the six-month follow-up period, which may be explained through the progression of wound healing. Following surgery, 24% of patients showed no HS-specific skin lesions after surgery and thus were considered as disease-free. However, a majority of patients in our study still had HS-specific skin lesions in the follow-up visits. This may be partly due to a multilocular disease manifestation but underlines the importance of a multimodal treatment approach in specific patients.

Interestingly, a subgroup analysis of patients who received split-thickness skin grafting after surgery did not show a significant improvement in postoperative quality of life or postoperative pain compared to secondary wound healing. There is only a limited number of studies addressing the topic of surgical reconstruction in HS surgery. Healing by secondary intention represents an established modality of wound healing in HS and is often considered as a first-line wound closure option providing excellent long-term outcomes and a low risk of recurrence [43]. Split-thickness skin grafts (STSGs) represent an alternative wound closure option in HS for the coverage of extensive surgical defects in all anatomic regions [8]. Several clinical studies have showed a shorter time to wound healing and a reduced risk of scar contraction in comparison to secondary intention healing; however, patient-reported outcome measures such as pain or quality of life were not considered in most studies [44].

This study has some limitations. There is only limited information regarding the inflammatory activity of the study patients through the use of relatively static disease severity scores. DLQI represents a validated but unspecific measurement tool for quality of life for patients with skin conditions [16]. Some HS-specific tools have been developed in recent years; however, their clinical use is limited and they were therefore not recorded in this study [20]. Treatment of HS comprises a combination of medical and surgical treatment strategies in a patient-oriented approach [8]. In this study, only patients naïve to systemic biologics were included and concomitant systemic medical therapies were not considered due to a lack of consistent information, which represents a limitation. Further studies are needed to investigate the impact of multimodal treatment regimens combining medical and surgical treatment strategies on patient-reported outcome measures.

In conclusion, this study visualized the marked impact of HS on the quality of life of affected patients. The restoration of everyday functionality represents a relevant endpoint in the treatment of HS from a psychosocial and socioeconomic perspective [45]. Surgical intervention marks an important pillar in the multimodal treatment of HS and is capable of significantly improving the quality of life of affected patients.

## Figures and Tables

**Table 1:** Personal and surgery-specific characteristics of HS patients. *n*, absolute number of patients; SD, standard deviation; y, years; IQR, interquartile range; BMI, body mass index; HS, hidradenitis suppurativa; HSS, Hidradenitits Suppurativa Score; DLQI, dermatologic life quality index; NRS, numerical rating scale.

Sex *n* (%)	female	42 (51.2)
	male	40 (48.8)
Age at surgery, mean (±SD), y		37.5 (±12.7)
BMI, mean (±SD); kg/m^2^		30.2 (±6.8))
Positive family history of HS, *n* (%)		27 (32.9)
Active smokers, *n* (%)		50 (61.0)
Age of HS onset, median (IQR), y		20.5 (10–60)
Prior treatments, *n* (%)	prior surgery	62 (75.6)
	prior antibiotics	51 (62.2)
Affected body regions, *n* (%)	Axilla	38 (46.3)
	Groins/genital	55 (67.1)
	Glutes/perianal	38 (46.3)
	Other	8 (9.8)
Surgery site, *n* (%)	Axilla	28 (34.1)
	Groins/genital	47 (57.3)
	Glutes/perianal	28 (34.1)
	Other	13 (15.8)
Hurley stages, *n* (%)	I	0 (0.0)
	II	66 (80.5)
	III	16 (19.5)
modified HSS, mean (±SD)		50.3 (±49.3)
DLQI, mean (±SD)		11.6 (±8.3)
Pain (NRS-11), mean (±SD)		3.01 (±3.04)

## Data Availability

The data that support the findings of this study are available on request from the corresponding author. The data are not publicly available due to privacy or ethical restrictions.
